# Peptide-based strategies for overcoming taxol-resistance in cancer therapy – a narrative review

**DOI:** 10.1007/s43440-025-00795-6

**Published:** 2025-10-20

**Authors:** Angelika Długosz-Pokorska, Katarzyna Gach-Janczak

**Affiliations:** Department of Biomolecular Chemistry, Faculty of Medicine, Medical University of Lodz, Mazowiecka 6/8, Łódź, 92-215, Poland

**Keywords:** Taxol, Cancer therapy, Drug resistance, Peptide-based therapies, Microtubule dynamics, Drug efflux

## Abstract

Taxol (Tx) is widely used in cancer therapy due to its ability to disrupt microtubule dynamics, inhibiting cell division and tumor proliferation. However, multidrug resistance (MDR) mechanisms, including enhanced drug efflux, altered metabolism, mutations in tubulin, and inhibition of apoptosis, challenge its efficacy. Peptide-based therapies have emerged as promising and significant solutions to overcome Tx resistance. These peptides offer high specificity, lower toxicity, and acceptable cell membrane penetration, enhancing precision medicine capabilities. Recent advancements focus on peptides that modulate drug efflux, stabilize microtubules, and promote apoptosis in cancer cells. The ATP synthase (ATP)-binding cassette transporters (ABC) transporter ABCB1, tubulin subunits, and anti-apoptotic proteins such as Bcl-2 show potential in restoring Tx effectiveness against MDR. Combining peptides with nanoparticle delivery systems improves tumor penetration and reduces side effects. Despite challenges like protease degradation and immunogenicity, peptide treatment addresses limitations. Peptide cancer therapies could revolutionize anticancer treatment by providing targeted, less toxic alternatives, especially for MDR. phenotype.

## Introduction

Taxol (Tx), also known as paclitaxel, is a significant drug in the treatment of various malignancies, including breast, ovarian, and non-small cell lung cancer. Its therapeutic efficacy primarily stems from its ability to disrupt microtubule dynamics, a process essential for cellular division, which is critical for the proliferation of cancer cells. Tx exerts its effects by stabilizing microtubule polymers, preventing their depolymerization, and inhibiting the formation of a mitotic spindle. This disruption inhibits proper chromosome alignment during mitosis, effectively arresting cell division and blocking the proliferation of tumor cells. However, the development of resistance to Tx represents a significant clinical obstacle, limiting its long-term therapeutic effects [1, 2].

Resistance mechanisms to Tx include alterations in drug metabolism, reduced cellular uptake, enhanced drug efflux *via* MDR transporters, and mutations in tubulin or associated proteins that disrupt microtubule stability and dynamics. These mechanisms collectively impair Tx’s ability to exert its effects. Therefore, it is essential to develop novel therapeutic strategies capable of overcoming these resistance mechanisms, to restore the Tx efficacy in cancer treatment [1, 2].

In recent years, peptide-based approaches have focused on their potential use in overcoming Tx resistance. Bioactive peptides offer several advantages over conventional small-molecule drugs. These advantages include high specificity for molecular targets in cancer cells, reduced drug toxicity, and enhanced cell membrane penetration [3, 4]. Peptides can exert their effects through various mechanisms, including receptor agonism or antagonism, as well as enzyme or protein-protein interaction/inhibition/modulation. The ability of these peptides to target specific pathways involved in cancer progression renders them powerful tools in the context of precision medicine. Furthermore, therapeutic peptides have potential oncology applications, including roles in hormone regulation and growth factor signaling. Peptides can be engineered to improve stability, extend circulation half-life, and enhance bioavailability, addressing issues related to potential immunogenic responses and rapid metabolic changes. Despite challenges like protease degradation and immunogenicity, peptide treatment addresses limitations [3–5].

This review aims to explore the mechanisms underlying Tx resistance and the potential of peptide-based strategies to improve Tx efficacy against MDR cancer phenotypes. Through a comprehensive examination of recent advancements and current research, this work explains how peptide-based therapies can be optimized for clinical application, ultimately advancing the field of targeted cancer therapies.

## Tx mode of action

Tx, a member of the taxane family, is a natural compound originally derived from the bark of the Pacific yew tree (*Taxus brevifolia*). Its anticancer properties were identified in the 1970s, several processes in cell division are disrupted, and the drug received approval in 1992 for the treatment of ovarian cancer. Moreover, Tx has proven effectiveness against various solid tumors, including breast and non-small-cell lung cancers [6, 7]. Tx interacts with tubulin, a cytoskeletal protein crucial for cellular processes. Unlike other tubulin-targeting drugs such as colchicine, which prevents microtubule assembly, Tx stabilizes microtubule polymers, inhibiting normal microtubule dynamics. This action significantly affects the separation of microtubules from centrosomes during mitosis [8–10]. Defects are observed in mitotic spindle assembly, chromosome segregation, and ultimately, cell division itself. Without proper spindle formation, chromosomes cannot align correctly during metaphase, causing a mitotic block. Prolonged activation of the mitotic checkpoint can inhibit apoptosis or cause cells to return to the G0 phase of the cell cycle [11]. The binding of Tx specifically to the beta-tubulin subunits of microtubules stabilizes them against depolymerization, allowing them to interfere with normal mitotic processes. This action on microtubule dynamics is key to Tx’s therapeutic activity, though it requires optimal conditions to achieve its efficacy [8, 12–15].

**Fig. 1:**
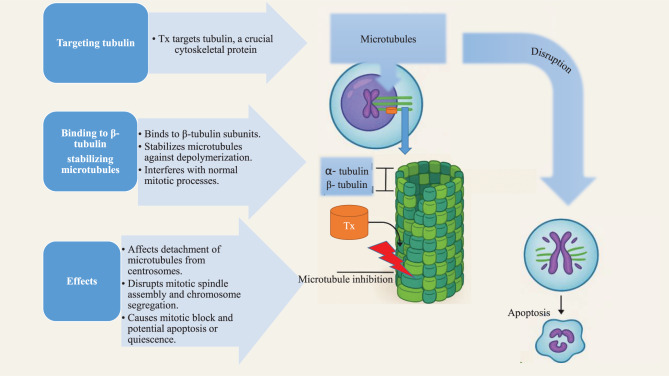
Mechanism of Tx action. Tx binds to beta-tubulin subunits of microtubules, stabilizing them against depolymerization and disrupting normal microtubule dynamics. This interference affects mitotic spindle assembly, chromosome alignment, and cell division, causing mitotic arrest and activating the mitotic checkpoint. These effects collectively inhibit proliferation and induce apoptosis in cancer cells. α-tubulin: alpha isoform of tubulin. β-tubulin: beta isoform of tubulin. Tx-Taxol

Understanding these mechanisms shows how Tx disrupts cell division and the cell cycle, highlighting its clinical significance in cancer treatment (Fig. 1).

## Mechanisms of Tx resistance

Resistance to Tx can occur through various mechanisms, which can be broadly categorized into pharmacokinetic and pharmacodynamic factors.

### Pharmacokinetic factors

#### Drug efflux pumps

The clinical efficacy of Tx can be compromised by resistance mechanisms, among which the increased expression of the ATP-dependent efflux pump ABCB1 (or MDR1) plays a significant role [16, 17].

ABCB1 encodes P-glycoprotein, a membrane transporter that actively pumps drugs out of cells, thereby reducing their intracellular concentration and efficacy. This efflux mechanism is well-documented in resistance to various chemotherapeutic agents, including chemotherapeutic drugs such as doxorubicin and Tx [16, 17].

Studies have linked resistance to Tx with increased ABCB1 expression, often resulting from chromosomal amplification events at 7q11.2–21 [18]. This amplification correlates with ABCB1 expression in Tx-resistant cells in many different cancer types, notably ovarian cancer [18]. It was demonstrated that several experimental models employing ABCB1 overexpression and ABCB1 inhibitors have validated the role of ABCB1 in promoting drug-resistant phenotypes [19]. The increased expression of murine homologs *Abcb1a* and *Abcb1b* has been shown in resistant models [20, 21]. Additionally, murine ATP transporters have been implicated in influencing the oral bioavailability and brain accumulation of drugs like rucaparib, suggesting broader implications for drug pharmacokinetics and efficacy [22].

The resistance to Tx linked to increased ABCB1 expression is acquired rather than innate. Chromosomal amplification and ABCB1 expression can arise as adaptive responses to drug exposure, promoting the survival and proliferation of resistant cancer cells under chemotherapy. Several experimental studies confirm that these changes result from selective pressure rather than a pre-existing genetic predisposition [18, 21, 22].

#### Metabolic inactivation

Enhanced expression of enzymes involved in drug metabolism, such as cytochrome P450 (CYP), can significantly contribute to the inactivation of Tx, thereby reducing its therapeutic efficacy. Among CYP enzymes, CYP2C8 plays a significant role in the metabolism of various drugs, including Tx. CYP2C8 exhibits substantial substrate specificity and metabolic activity against a broad spectrum of drugs. Notably, it metabolizes antidiabetic agents like rosiglitazone and pioglitazone, the antiarrhythmic drug amiodarone, and the natural anticancer agent Tx [23].

Several studies have demonstrated that genetic variants of CYP2C8 can influence its metabolic activity towards Tx [24]. For instance, the CYP2C8 × 3 variant, characterized by the R139K and K399R mutations, which is more prevalent in white populations, significantly reduces metabolic activity towards Tx. This variant shows up to an 80% decrease in clearance rates compared to the wild-type enzyme [24]. Moreover, heterologous expression studies have shown that variants like CYP2C8.2 present altered kinetic parameters, including a higher Michaelis constant (Km), resulting in a two-fold decrease in intrinsic clearance for Tx compared to the wild-type enzyme [23–27].

### Pharmacodynamic factors

Pharmacodynamic mechanisms of Tx resistance are related to alterations at the cellular and molecular levels, particularly in how the drug interacts with its target microtubules [2, 28, 29], [30].

Microtubules are dynamic, tube-like structures composed of α- and β-tubulin dimers that are significant to maintaining cell shape and facilitating processes such as mitosis. Tx specifically binds to β-tubulin, stabilizing microtubules and preventing their depolymerization during cell division. However, alterations in microtubule polymerization and depolymerization dynamics can reduce the effectiveness of drug treatment [2, 28–30]. Microtubules are highly dynamic polymers that undergo continuous phases of growth and depolimerization, a phenomenon referred to as dynamic instability. Tx exerts its cytotoxic effect by binding to β-tubulin, thereby stabilizing microtubules. Finally it prevents microtubule disassembly during mitosis. In several cancers, tumor cells may acquire specific tubulin variants, such as βIII-tubulin, which exhibit reduced affinity for Tx. The presence of these mutations or isotype shifts is frequently associated with poor prognosis and diminished therapeutic response [2]. However, in Tx-resistant cells, alterations in the rates of microtubule polymerization and depolymerization, often driven by mutations in tubulin or dysregulation of MAPs [2]. It regulates microtubule dynamics by binding to their surface, modulating stability, and influencing spatial organization. In normal cells, MAPs such as tau and MAP4 contribute to the regulation of microtubule dynamic instability. However, in Tx-resistant cancer cells, overexpression of specific MAPs can antagonize the drug’s stabilizing effect, leading to microtubules that remain excessively stable and less responsive to Tx. This sustained stability preserves microtubule integrity despite drug exposure, thereby diminishing Tx efficacy and promoting resistance. Such changes compromise Tx efficacy by rendering microtubules less stable and less responsive to drug-induced stabilization [28, 29]. Tubulin is subject to diverse post-translational modifications (PTMs), including acetylation, detyrosination, and polyglutamylation, which collectively modulate microtubule stability and dynamics. In Tx-resistant cells, alterations in these PTMs can impair drug efficacy. For example, increased acetylation or detyrosination enhances microtubule stability and reduces their susceptibility to Tx-mediated stabilization. As a result, microtubules resist destabilization even in the presence of the drug, thereby contributing to resistance [30]. The key pharmacodynamics mechanisms include changes in microtubule dynamics, mutations in tubulin, modifications in the expression of microtubule-associated proteins (MAPs), variations in tubulin isotype composition, and PTMs of tubulin [2, 28–30] (Table 1).

**Table 1:** Key mechanisms contributing to tx treatment resistance in cancer cells

Mechanism	Impact on cancer cells	Cell type/line	Reference
Alterations in microtubule dynamics	Microtubules are dynamic structures that alternate between growth and shrinkage, a process known as dynamic instability. Tx binds to β-tubulin, stabilizing microtubules and preventing disassembly during mitosis. In Tx-resistant cells, changes in the rates of microtubule polymerization and depolymerization, or improper regulation of microtubule dynamics, occur. These alterations, which may result from mutations in tubulin or changes in MAPs, reduce microtubule stability and responsiveness to Tx, thereby decreasing the drug’s efficacy.	CHO, HeLa, A549–T12, T-24	[2]
Mutations in tubulin	Tubulin mutations can alter the structure of α- and β-tubulin, which make up microtubules, affecting the binding affinity of Tx for its target sites. These mutations reduce the ability of Tx to bind effectively, thereby decreasing its capacity to stabilize microtubules during mitosis and contributing to the development of drug resistance. In some cancers, tumor cells may express specific tubulin variants, such as βIII-tubulin, which have lower affinity for Tx. Certain mutations are cancer-type specific, and the presence of particular tubulin variants can indicate poor prognosis or resistance to Tx.	CHO, HeLa, A549–T12, T-24, MDA-MB231/K20T	[2]
Expression of MAPs	MAPs regulate microtubule dynamics by binding to microtubules and promoting their stability or affecting their organization. In normal cells, MAPs such as tau and MAP4 control the dynamic instability of microtubules. In Tx-resistant cancer cells, overexpression of specific MAPs can maintain microtubule integrity even in the presence of Tx, counteracting the drug’s stabilizing effects. As a result, Tx’s primary mode of action is disrupted, reducing its efficacy and contributing to drug resistance.	ALL, NSCLC	[28]
Tubulin isotype composition	The expression of βIII-tubulin, a specific β-tubulin isotype, has been shown to correlate with Tx resistance in several cancers. This isotype displays a lower binding affinity for Tx, resulting in reduced drug efficacy. Such a shift in tubulin isotype expression decreases Tx binding and compromises microtubule stabilization, ultimately contributing to drug resistance.	MCF-7, SUIT 2, NSCLC, Ovarian (Clear cell adenocarcinoma)	[29]
Post-translational modifications of tubulin	In resistant cells, alterations in tubulin PTMs, such as increased acetylation or detyrosination, can enhance microtubule stability and reduce their susceptibility to the stabilizing effects of Tx. As a result, microtubules remain resistant to destabilization despite the presence of the drug, thereby diminishing Tx efficacy.	MKN45, MCF-7, HeLa, ELC17	[30]

**Abbreviation list** A549–T12,*T*-24- Tx resistance adenocarcinomic human alveolar basal epithelial cells. ALL cells-Acute lymphocytic leukemia. β-tubulin – Beta isoform of tubulin

βIII-tubulin- Beta III isoform of tubulin. CHO-Resistant cells Chinese hamster ovary. ELC17-Patient-derived primary lung cancer. HeLa- Human cervical carcinoma cells. MAPs- Microtubule-associated proteins. MCF-7-Human breast cancer cell line. MDA-MB 231/K20T -The model of late-stage triple-negative breast cancer. MKN45 -Human gastric cancer cell line. NSCLC cells-Non-small-cell lung cancer. SUIT 2-Pancreatic carcinoma cell line. Tx-Taxol

### Apoptosis evasion

Resistance to Tx, a microtubule-stabilizing agent, often involves inhibition of apoptosis mediated by dysregulation of the activity of BCL-2 family proteins. This section explores how targeting downstream members of the apoptotic pathway could offer effective strategies against Tx resistance.

#### Role of BCL-2 family proteins in apoptosis evasion

The BCL-2 family proteins, including Bcl-XL and Bcl-2, play critical roles in regulating apoptosis by controlling mitochondrial membrane permeability and the release of cytochrome c [31, 32]. These proteins bind to pro-apoptotic members such as Bax and Bak, creating heterodimers that inhibit apoptosis. A disruption of the expression ratio of anti-apoptotic (Bcl-XL/Bcl-2) to pro-apoptotic (Bax) proteins can suppress apoptosis, contributing to MDR phenotype [31, 32].

For instance, scientists proved that in ovarian cancer, overexpression of Bcl-XL and Bcl-2 has been linked to resistance against Tx-induced apoptosis. Genetic variants of Bcl-2 have also been associated with increased resistance to Tx treatment [33, 34].

Strategies aimed at disrupting the binding of Bcl-XL and Bcl-2 to pro-apoptotic proteins offer promising solutions to overcome apoptosis evasion in Tx-resistant cancers. For example, small interfering RNA (siRNA) targeting Bcl-XL has been shown to decrease cell survival and sensitize cancer cells to chemotherapy, demonstrating a therapeutic approach to restoring apoptosis sensitivity [35, 36]. Approaches targeting the modulation of Bcl-2 and Bcl-XL expression and activity have been shown to hold considerable potential in overcoming resistance by sensitizing cancer cells to Tx-induced apoptosis [35, 36].

## Peptide-based strategies to overcome MDR resistance

Peptide-based therapies have been developed as a promising strategy to combat Tx resistance due to their specificity, versatility, and relatively low toxicity. The following sections discuss various peptide-based approaches to enhance the efficacy of Tx in resistant cancer cells.

### Peptides targeting drug efflux pumps

#### Inhibitory peptides

Peptides targeting drug efflux pumps represent a promising approach to overcoming resistance to anticancer drugs such as Tx. In peptide-based strategies, inhibitory peptides like HX-12C have demonstrated significant therapeutic potential. Studies have shown that the HX-12C peptide demonstrates different dynamic structural properties depending on its environment. In aqueous solutions, it adopts a random coil structure, whereas in membrane-like and acidic solutions, it adopts a helical conformation. This structural adaptability enhances its anticancer activity because it correlates with the peptide’s ability to interact with cellular membranes [3].

Cytotoxicity studies have shown that peptide HX-12C combats resistant cell lines, achieving efficacy similar to that in non-resistant cell lines. Notably, peptide HX-12C is particularly effective in sensitizing ABCB1-overexpressing cells to ABCB1 substrates such as Tx and doxorubicin. Importantly, it does not affect sensitivity to cisplatin, indicating specificity for ABCB1-mediated resistance mechanisms [3].

Furthermore, mechanistic studies have provided insights into the activity of HX-12C. Rather than altering the expression or cellular localization of the ABCB1 protein, HX-12C inhibits ABCB1 transporter function. HX-12C enhances the intracellular accumulation of Tx in ABCB1-overexpressing cells, similar to the effect of verapamil, a known ABC transporter inhibitor [3].

#### Peptide-nanoparticle

Peptide-nanoparticle (NP) cancer therapies are emerging as a promising approach for cancer treatment, offering targeted therapy and monitoring capabilities. Various nanoparticles are modified with peptides to enhance their specificity for cancer cells, thereby reducing damage to healthy tissue. These peptide-particles are administered to cancer cells by mechanisms such as endocytosis, leading to cellular damage and activation of apoptosis pathways [37–39]. Despite their potential, only a limited number of peptide-NP therapies have received clinical approval, largely due to low specificity for cancer cells. However, recent research has focused on optimizing NP functionalization for cancer treatment, including advancements in self-assembling peptide nanostructures with enhanced stability and bioavailability [37–42].

Yakati et al. [43] demonstrated that poly (lactic-co-glycolic acid) (PLGA) nanoparticles were conjugated with the tumor-targeting peptide CPKSNNGVC (CPK) and Tx using maleimide-thiol chemistry. This combination showed a high affinity for the monocarboxylate transporter 1 (MCT1) receptor, which is overexpressed in colorectal cancer cells. Scientists demonstrated a significant reduction in new blood vessel formation, highlighting the potential of this nano-drug as an effective treatment for colorectal cancer [43].

Zaman et al. [44] presented a novel nanoplatform comprising self-assembling spherical nanoparticles derived from naphthalene diamide and surface-modified with transferrin, a ligand targeting transferrin receptors, which are overexpressed in breast and cervical cancer cells. These nanoparticles were loaded with Tx and possessed intrinsic fluorescence properties, enabling their application in both drug delivery and imaging. The system exhibited high stability and facilitated a sustained release profile, with over 90% of the drug released under both physiological and acidic conditions. Crucially, the formulation selectively triggered apoptosis in cancer cells, offering superior antitumor activity and lower toxicity compared to the free drug [44].

### Peptides modulating microtubule dynamics

**Fig. 2:**
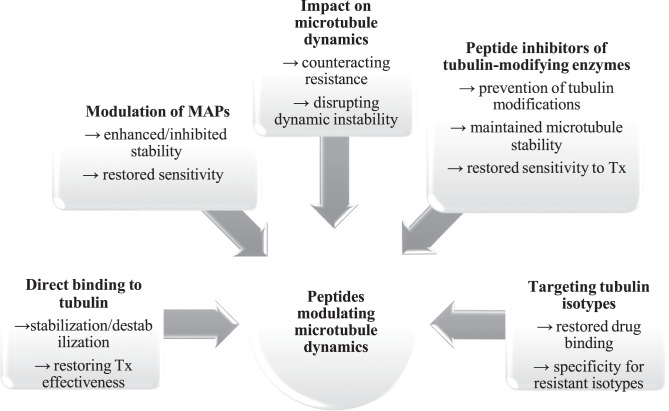
Anticancer peptide-induced modulation of microtubule dynamics and drug resistance.Specific anticancer peptides interact with tubulin subunits to alter microtubule polymerization and depolymerization, disrupt mitotic progression, enhance chemotherapy effectiveness, and counteract drug resistance by inhibiting efflux transporters in resistant cancer cells. MAPs- microtubule-associated proteins. Tx-Taxol

Peptides have proven to be powerful agents in cancer therapy, especially in addressing drug resistance related to microtubule dynamics. By directly binding to tubulin, modulating MAPs, and influencing post-translational modifications, peptides play a crucial role in regulating microtubule stability [10, 45–51]. Modulation of MAPs can enhance or inhibit microtubule stability, thereby restoring cellular sensitivity to Tx. By influencing microtubule dynamics, such peptides can counteract resistance mechanisms and disrupt abnormal dynamic instability. Another approach involves peptide inhibitors of tubulin-modifying enzymes, which prevent post-translational modifications and help maintain microtubule stability, leading to renewed Tx responsiveness. In addition, anticancer peptides capable of directly binding tubulin may stabilize or destabilize microtubules as needed, reinforcing Tx effectiveness. Targeting specific tubulin isotypes with selective peptides represents a further strategy, as it restores drug binding affinity and addresses resistance linked to the expression of resistant isotypes. Together, these mechanisms highlight the therapeutic potential of anticancer peptide-based modulators in overcoming Tx resistance. (Fig. 2).

#### Direct binding to tubulin

Peptides that bind directly to tubulin can significantly change the rate of polymerization and depolymerization in microtubules. During the binding to tubulin, these peptides can stabilize and/or destabilize microtubules, depending on the specific binding site on the tubulin in cancer cells. This mechanism is similar to the action of Tx, which stabilizes microtubules by binding to β-tubulin. In Tx-resistant cancer cells, peptides that bind tubulin and stabilize microtubules can help achieve the drug effectiveness [45, 46]. For instance, stathmin obtained from natural tubulin-binding proteins has been shown to stabilize microtubules and inhibit cell division in cancer cells, overcoming Tx resistance [10].

#### Modulation of MAPs

Peptides can modulate the function of MAPs, which play a key role in maintaining microtubule stability and dynamics. Changing MAP activity, peptides can either promote or disrupt microtubule stability, thereby impacting cellular responses to Tx [45]. The regulation and changes in MAPs expression are linked to mechanisms of resistance. Peptides targeting MAPs can help restore sensitivity to Tx, making cancer cells more susceptible to treatment. Several studies demonstrated that changes in the expression of different isoforms of the MAP4 have been shown to affect the sensitivity of cancer cells to microtubule-targeting agents. In Tx-resistant ovarian cancer cell lines, MAP4 phosphorylation and its detachment from microtubules were associated with reduced sensitivity to Tx [46, 47].

#### Impact on microtubule dynamics

Microtubules are characterized by dynamic instability, meaning they undergo rapid cycles of growth and shrinkage, a process that is essential for cell proliferation. Some peptides can stabilize microtubules by a mechanism similar to Tx, thereby enhancing their effects. Alternatively, peptides can interfere with the dynamic instability of microtubules, disrupting the processes of growth and shrinkage to counteract resistance mechanisms [45–47].

Furthermore, Tau-derived peptides can stabilize microtubules, and they have shown promise in overcoming Tx resistance. These peptides bind to tubulin and enhance microtubule stability. This mechanism of action is particularly beneficial in cancers that have developed resistance through alterations in microtubule dynamics [45].

#### Targeting tubulin isotypes

Tubulin, a key structural component of microtubules, exists in multiple isotypes, each with unique properties influencing its interaction with therapeutic agents such as taxanes [48, 49]. In resistant cancer cells, the tubulin isotypes change their forms with reduced affinity for Tx. Notably, the β-tubulin isotypes have been identified as a significant contributor to Tx resistance. Moreover, its overexpression is observed in advanced cancers and correlates with a diminished response to Tx therapies. This isotype interferes with Tx-induced microtubule assembly, undermining the drug’s stabilizing effects on the microtubule network [50]. Several recent studies demonstrated the potential of peptides specifically designed to target resistant-associated tubulin isotypes like βIII-tubulin. These peptides can modulate microtubule dynamics, enhancing the affinity of Tx for tubulin and restoring its therapeutic efficacy. For instance, peptides that selectively bind βIII-tubulin can counteract its destabilizing effects, promote microtubule stability, and improve drug efficacy. By directly addressing the MDR phenotype in cancer cells, these peptides provide a novel mechanism to overcome the limitations posed by isotype-specific drug interactions [48–50].

#### Peptide inhibitors of tubulin-modifying enzymes

Another promising approach to overcoming Tx resistance involves the use of peptides that inhibit tubulin-modifying enzymes [10, 50]. These enzymes, such as tubulin acetyltransferase, play a significant role in regulating post-translational modifications of tubulin, leading to microtubule stability and dynamics. In resistant cancer cells, tubulin modifications can destabilize microtubules or modify their dynamics, ultimately reducing the sensitivity to Tx. Peptides specifically designed to inhibit these enzymes can block such tubulin modifications, thereby improving microtubule stability and restoring the cytotoxic effects of Tx. a connection between tubulin acetylation and Tx sensitivity, particularly in lung cancer. Elevated levels of acetylated tubulin have been linked to increased resistance to Tx-induced cell death. Moreover, tubulin acetylation enhances the stability of the anti-apoptotic protein Mcl-1 by protecting it from proteasome degradation, which promotes cell survival in the presence of Tx. By targeting tubulin acetyltransferase or similar modifying enzymes, therapeutic peptides can reduce tubulin acetylation, destabilize Mcl-1, and re-sensitize cancer cells to Tx-induced apoptosis [51].

The strategy of using peptide inhibitors of tubulin-modifying enzymes holds significant potential for improving outcomes in Tx-resistant cancers. By preventing the biochemical alterations that weaken Tx efficacy, enzyme-targeting peptides represent a valuable addition to the arsenal of resistance-mitigating therapies [10, 51].

### Peptides targeting apoptotic pathways

Pro-apoptotic peptides, behaving similarly to the endogenous pro-apoptotic proteins, represent a promising approach to sensitize cancer cells to Tx-induced apoptosis [52]. These peptides act by enhancing mitochondrial outer membrane permeabilization (MOMP), a critical step in the intrinsic apoptotic pathway. For example, peptides derived from the BH3 domain of Bcl-2 family proteins can promote MOMP and trigger apoptosis, improving the effects of Tx action. By changing pro-apoptotic signaling, these peptides amplify the cell death mechanisms initiated by Tx, particularly in cancer cells that have developed resistance to the drug [52]. In addition to promoting apoptosis, peptides that inhibit anti-apoptotic proteins offer a second strategy to enhance Tx efficacy. Anti-apoptotic members of the Bcl-2 family, such as Bcl-2 and Bcl-XL, sequester pro-apoptotic proteins like Bax, preventing the activation of apoptosis. Peptides that disrupt these interactions have shown promise in preclinical models by restoring the apoptotic response in Tx-resistant cells [53, 54]. For instance, by targeting the Bcl-2/Bax interaction, these peptides can effectively promote apoptosis and overcome resistance mechanisms in cancer cells. One such compound, NuBCP-9, a highly promising anticancer peptide, specifically induces apoptosis in cancer cells by exposing the BH3 domain of Bcl-2, thereby inhibiting the survival function of Bcl-XL [52]. NuBCP-9 has been shown to induce Bcl-2-mediated apoptosis in breast and lung cancer cell lines, leading to the hypothesis that it may also reverse Bcl-2-mediated resistance to Tx [54].

### Combination therapies - optimizing peptide stability

The application of combination therapies involving Tx and the optimization of peptide stability holds substantial promise in overcoming drug resistance in cancer treatment [55]. Tumor-penetrating peptides, such as iRGD, have emerged as powerful tools in advanced drug delivery systems due to their ability to enhance targeted drug delivery [54]. The iRGD peptide, in particular, stands out for its tumor-targeting ability [55, 56]. By binding to integrin on tumor cells, iRGD initiates a transcytosis pathway that enables deep tumor penetration. and good action. In combination with Tx in nanoparticle systems, iRGD can guide the drug deep into tumor tissues, effectively addressing one of the major obstacles in cancer therapy: the penetration of dense tumor structures. This combination enhances Tx efficacy by passing biological barriers and concentrating the drug within the tumor, resulting in more effective and less toxic treatment [57–60].

The stability of peptides, which is crucial to their function in combination therapies, is enhanced by modifications like PEGylation and cyclization, which prevent enzymatic degradation and extend the peptides’ half-life in circulation. This stability is critical for enabling controlled, sequential release mechanisms within nanoparticle formulations. Additionally, the incorporation of peptidomimetics and non-natural amino acids—such as D-amino acids, β-amino acids, and backbone-modified residues—further improves proteolytic resistance, enhances structural rigidity, and prolongs systemic exposure, making these engineered peptides more suitable for therapeutic applications in oncology [61]. Through these systems, ABCB1 inhibitors can first destabilize efflux pumps, allowing subsequent Tx release to maximize intracellular retention and effectiveness. The precision during this modification not only improves the potency of the therapeutic approach but also minimizes the likelihood of resistance development by maintaining optimal drug levels at the tumor site for a sustained period [62, 63].

Preclinical studies have shown that peptide-based combination therapies improve drug delivery and decrease toxicity during treatment, leading to better therapeutic outcomes [64]. Scientists have demonstrated that using lipid-polymer nanoparticle (LPN) systems to co-deliver peptides with chemotherapeutic agents results in prolonged action within cancer cells and enhanced intracellular retention. These combination therapies, which demonstrate improved targeting and minimized side effects, are currently being evaluated in clinical trials for various cancer types to translate preclinical achievements into effective patient treatments [64].

To enhance Tx delivery, researchers developed liposomes functionalized with TAT peptide and cleavable PEG via a redox-sensitive linker (PTX-C-TAT-LP). In circulation, PEG shields TAT, prolonging blood half-life. At the tumor site, elevated glutathione (GSH) removes PEG, exposing TAT and boosting cellular uptake. This system showed improved tumor penetration, enhanced distribution, and a 69.4% inhibition rate in murine melanoma models, with no significant liver or heart toxicity. PTX-C-TAT-LP represents a promising tumor-targeted delivery platform [65].

Another approach involves membrane-disrupting peptides (MDPs), a promising class of therapeutic agents capable of overcoming Tx resistance in cancer treatment. These peptides selectively interact with the negatively charged membranes of cancer cells, causing membrane destabilization, pore formation, and ultimately cell lysis. Recent studies have shown that antimicrobial peptides involving MDP peptides with anticancer properties, including cationic amphipathic peptides, exhibit synergistic effects when combined with chemotherapeutics, enhancing drug uptake and bypassing efflux mechanisms [66].

### Clinical translation of peptide-based strategies in tx-enhanced anticancer therapy

Peptide-based therapeutic strategies have shown considerable promise in preclinical studies. However, their clinical translation remains limited, despite their significant potential. Ongoing investigations into peptide-drug conjugates (PDCs) and peptide-functionalized nanoparticle systems aim to enhance the targeted delivery and therapeutic efficacy of Tx [67]. Several studies have shown that a completed Phase I clinical trial (Identifier: NCT00666991; Registration Date: April 23, 2008) assessed the safety and pharmacokinetics of intraperitoneally administered nanoparticulate Tx (Nanotax), demonstrating the potential of peptide-modified carriers to improve drug distribution while minimizing systemic toxicity [67, 68]. Despite these developments, significant challenges remain, including the enzymatic instability of peptides in vivo, restricted tissue penetration, potential immunogenic responses, and difficulties associated with large-scale manufacturing. Technologies such as covalent, peptide-based lysosome-targeting platforms (Pep-TACs) present innovative approaches to overcoming these limitations by facilitating enhanced intracellular delivery and degradation of cancerogenic proteins. A thorough understanding of these translational barriers is very important to fully realize the therapeutic potential of peptide-based cancer therapy [67, 68].

## Conclusion

In conclusion, Tx remains a cornerstone in the treatment of various cancers due to its potent ability to disrupt microtubule dynamics, thereby impeding cell division and halting the proliferation of rapidly dividing tumor cells. However, the development of resistance to Tx is a significant clinical hurdle that compromises its therapeutic efficacy. Resistance mechanisms, including enhanced drug efflux, altered metabolism, mutations in tubulin, and evasion of apoptosis, necessitate the exploration of new strategies to enhance the drug’s effectiveness. The emergence of peptide-based therapies offers a promising direction for overcoming Tx resistance. Peptides have distinct advantages over traditional chemotherapeutic agents, including their high specificity, lower toxicity to normal cells, and ability to penetrate cell membranes efficiently. Furthermore, peptides can be engineered to target specific molecular pathways involved in cancer progression, making them ideal candidates for precision medicine.

**Fig. 3:**
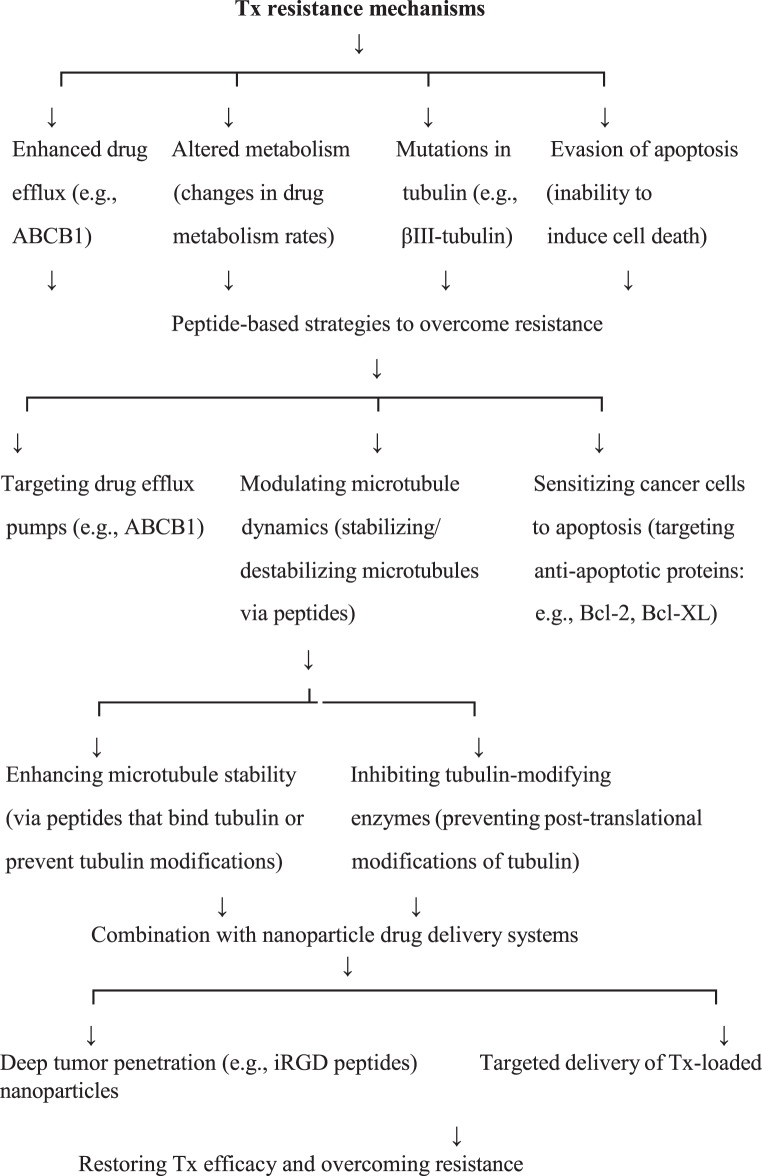
Strategies involving anticancer peptides to overcome tx resistance in cancer therapy. Anticancer peptides restore tx sensitivity and improve anticancer efficacy by regulating microtubule dynamics, inhibiting drug efflux transporters such as P-glycoprotein, and modulating apoptosis and survival signaling pathways in resistant tumor cells. ABCB1: ATP-binding cassette subfamily b member 1. Bcl-2: B-cell lymphoma 2. Bcl-XL: B-cell lymphoma-extra large. βIII-tubulin: beta III isoform of tubulin. iRGD: internalizing RGD peptide. Tx – taxol

Recent advancements in peptide-based strategies, including the development of peptides that target drug efflux pumps, modulate microtubule dynamics, and sensitize cancer cells to apoptosis, have demonstrated significant potential in improving the efficacy of Tx in resistant cancer phenotypes. By targeting specific resistance mechanisms, such as the overexpression of ABCB1, peptide inhibitors can restore Tx effectiveness in overcoming drug resistance. Moreover, peptides that interact with tubulin or inhibit tubulin-modifying enzymes can enhance microtubule stability, complement the Tx mode of action, and make cancer cells more susceptible to its effects. Furthermore, the development of peptides that modulate apoptotic pathways, particularly those that target anti-apoptotic proteins like Bcl-2 and Bcl-XL, has shown promise in reversing apoptosis evasion, a key mechanism of resistance in Tx-treated cancer cells. Peptide-based therapies also hold potential in combination with other drug delivery systems, such as nanoparticle technologies, which can improve the stability, bioavailability, and targeted delivery of chemotherapeutic agents. For example, peptides like iRGD, which facilitate deep tumor penetration, can be coupled with Tx-loaded nanoparticles to overcome biological barriers and enhance drug accumulation at the tumor site. This targeted approach not only improves therapeutic outcomes but also minimizes off-target toxicity, thereby enhancing the overall safety profile of cancer treatments. Despite the promise of peptide-based strategies, challenges such as protease degradation, immunogenicity, and limited clinical application remain. However, ongoing research efforts focused on optimizing peptide stability, delivery methods, and combinatorial therapies are addressing these limitations. The integration of peptide-based approaches into clinical practice holds the potential to revolutionize cancer treatment by providing more effective, targeted, and less toxic therapies, particularly for patients with multidrug-resistant cancers (Fig. 3).

## Data Availability

This is a review article. Data sharing is not applicable, as no datasets were generated or analyzed during the current study.

## Abbreviation

7q11.2–21: Chromosomal region 7q11.2–21
ABC transporters: ATP synthase (ATP)-binding cassette transporters
ABCB1 (MDR1): ATP-binding cassette sub-family B member 1 (Multi-Drug Resistance protein 1)
ABCB1A/ABCB1B: Murine homologs of ABCB1
Bak: Bcl-2 homologous antagonist/killer
Bax: Bcl-2-associated X protein
Bcl-2: B-cell lymphoma 2
Bcl-XL: B-cell lymphoma-extra large
BH3 domain: Bcl-2 homology 3 domain
CPK: Tumor-targeting peptide CPKSNNGVC
CYP: Cytochrome P450
CYP2C8: Cytochrome P450 2C8 isoform
CYP2C8 × 3: CYP2C8 variant *3
GSH: Glutathione
iRGD: internalizing RGD peptide
Km: Michaelis constant
LPNs: Lipid-polymer nanoparticles
MAP4: Microtubule-associated rotein 4
MAPs: Microtubule-associated proteins
MCT1: Monocarboxylate transporter 1
MOMP: Mitochondrial outer membrane permeabilization
NP: Nanoparticle
NuBCP-9: Nuclear Bcl-2 converting peptide-9
PDCs: Peptide-drug conjugates
PEG: Polyethylene glycol
PEGylation: Polyethylene glycol modification
Pep-TACs: Peptide-based Lysosome-targeting chimeras
PLGA: Poly(lactic-co-glycolic acid)
PTMs: Post-translational modifications
PTX-C-TAT-LP: Paclitaxel-cleavable-TAT-liposome
siRNA: Small interfering RNA
TAT peptide: Trans-Activator of transcription peptide
Tx: Taxol
